# The Dimensions of Multiple Chronic Conditions: Where Do We Go From Here? A Commentary on the Special Issue of *Preventing Chronic Disease*

**DOI:** 10.5888/pcd10.130104

**Published:** 2013-04-25

**Authors:** Robert B. Wallace, Marcel E. Salive

**Affiliations:** Author Affiliation: Marcel E. Salive, Division of Geriatrics and Clinical Gerontology, National Institute on Aging, Bethesda, Maryland.

The articles in this issue address the high prevalence and substantial clinical burden of multiple chronic conditions (MCC) among adults. All of these papers further the goals outlined in the US Department of Health and Human Services (DHHS) MCC Strategic Framework (1,2). The article by Goodman, Posner, Huang, Parekh and Koh (3) introduces the topic and describes the origin of the 20 conditions originally selected by the DHHS for emphasis. The authors also provide a conceptual model for standardizing data approaches to the analyses of MCC. The remaining articles document various distributions and rates of MCC on the national level with analyses of important federal health surveys and databases: Lochner and Cox analyzed Medicare claims data (4); Ashman and Beresovsky analyzed 1 year of the National Ambulatory Medical Care Survey (5); Ford, Croft, Posner, Goodman, and Giles explored the prevalence of lifestyle-related MCC from the National Health Interview Survey (6); Steiner and Friedman examined MCC-related acute care hospitalization rates from the Nationwide Inpatient Sample of the Healthcare Cost and Utilization Project (7); Soni and Machlin analyzed the costs of certain MCC from the Medical Expenditure Panel Survey (8), and Ward and Schiller estimated MCC rates from the National Health Interview Survey (9).

Each of these articles explores different data sources, and despite the variation in disease and condition combinations selected, these articles show the ability of many US federal datasets to address and better characterize the scope of MCC as well as incorporate important MCC-related issues such as the effect of MCC on the cost of clinical care and the extent of clinical care use. Collecting data from multiple sources, including population surveys and claims data, and from both institutional settings and ambulatory primary care allows triangulation and better comprehension of this issue. Although the challenges of the complex MCC patient have long been recognized, these articles highlight national prevalence rates and implications for prevention, diagnosis, management, and important outcomes. Here we suggest some directions for addressing MCC in the future and offer suggestions on how to address this complexity on the basis of the work presented in this issue and the growing body of emerging information on MCC.

## Defining Diseases in the Context of MCC

A detailed exposition on defining a distinct disease or condition is beyond the scope of this discussion, but important considerations abound. Here we examine some MCC-relevant issues related to such definitions. In this issue, Goodman et al have contributed importantly to solving this problem (3), and even the terms used to describe MCC have varied (10); however, many pragmatic questions remain.

Defining a chronic condition and MCC requires careful consideration. Many have defined such conditions on the basis of duration as conditions lasting at least 6 to 12 months; in the DHHS Framework (1), a chronic condition is defined as a condition lasting 12 or more months and requiring ongoing medical care. But how should remittent diseases such as asthma, certain mental illnesses, or multiple sclerosis be considered? What about late recurrences of tumors thought to be controlled? In our view, period prevalence rates are not sufficient.

Another issue is whether to consider many infectious diseases as chronic conditions. Goodman et al (3) note the chronic nature of HIV infection, but other important chronic infections exist, such as tuberculosis and hepatitis B and C. In our view these conditions, and their co-occurring illnesses, encumber all of the management challenges of important noninfectious diseases such as coronary heart disease, cancer, diabetes, or stroke-related disability (11,12). These issues dovetail with the challenges of defining MCC, which are highlighted in the articles in this issue and in other articles (13–15).

How do you deal with complex clinical manifestations of conditions, such as signs (visually observable patient abnormalities), symptoms (abnormal perceptions of illness that only the patients can report, such as pain, itching, fatigue, depressive feelings), and syndromes (clusters of signs, symptoms, and other clinical phenomena that may or may not be indicative of a specific underlying disease)? Do these belong in the study of MCC? Our answer is that these signs, symptoms, and syndromes must be carefully and systematically addressed, since many never reach the level of a specific diagnosable “disease” with an ICD code; however, they can cause considerable suffering and require health care .

An “individual” disease — is it one or many? Many diseases that are regarded as single entities exhibit diverse organ involvement and, over time, special and distinct clinical manifestations and sequellae. For example, diabetes mellitus is clearly associated with coronary heart disease, renal insufficiency, retinal disease, skin abnormalities, and other important clinical problems. Should each of these be considered separately in the multiplicity of MCC or as part of 1 condition for analytical purposes? Again, it depends on the question being addressed.

How should the “secondary outcomes” of a variety of biologically unrelated chronic conditions be considered and counted? Many chronic conditions clearly lead to a variety of unfortunately common and functional outcomes that are not necessarily related to the underlying causes or pathogenesis of the primary disease, including falls, cognitive impairment, anemia, malnutrition, polypharmacy, sleep disorders, and sexual dysfunction. Among older persons, some of these conditions have been called “geriatric syndromes” (16). Often, statistically significant associations between various primary index illnesses and these secondary outcomes are present, even if the latter are not biologically related to the primary condition. The complex downstream pathways for additional chronic illnesses, whether they are biologically related or less specific secondary conditions, may all be clinically important; preventive interventions may be as important as managing the primary condition (17).

In counting diseases and conditions, at least 2 other issues remain. First, how should adverse effects of therapy be counted; they can be costly and deadly (18). Second, how should disease risk factors such as hypertension or hypercholesterolemia be considered, and the physiological changes of aging, such as osteopenia or sarcopenia? These “nondiseases” require further consideration as MCC (19,20).

## Data Quality in Studying MCC

There are many issues that impinge on the quality of data used to assess MCC that relate to all population data on disease occurrence, as well as to their risk factors, prevention, treatment, and outcomes. Here are some of the central ones:

### Taxonomy and nomenclature

Maintaining a consensual, standard nomenclature and taxonomy is, of course, critical for quantifying diseases and conditions in community and health care settings, and equally critical for counting disease co-occurrences and permutations of MCC as well as understanding their outcomes (21). This is particularly important for both health insurance claims data and clinical records, because of variation in medical terminology, so-called “natural language,” and coding practices. The International Classification of Diseases (ICD) provides a standard nomenclature for most medical conditions (22).

New research on taxonomy and nomenclature will not only change the taxonomic systems currently in place; it will improve the understanding of disease causation and management. An important example of this is the ability to empirically cluster various individual diseases in terms of how “close” they are to each other with respect to their known pathogenic mechanisms (23), thus allowing a perspective on the preventability of various combinations of MCC. This may lead to some possible “lumping” of conditions that may seem heterogeneous but have common causes and possible common management. This is contrary to current taxonomic activities, where more basic science has led to more “splitting” (disaggregation) of diseases into more and finer diagnostic rubrics.

### Medical care access and disease surveillance

Not all persons with diseases have full and equal access to medical care, and thus some diseases and conditions are never identified. Even where full care access is present, professional variation in disease screening, diagnosis, and treatment will occur among individual practitioners and across health care institutions and systems (24). Another source of variation in MCC, so-called “diagnostic bias,” may occur because clinically managing one illness may increase the likelihood that another will be identified as a result of more frequent exposure to medical services (25). In MCC analytical studies, disease ascertainment may be enhanced for certain chronic conditions by searching for certain prescription drug or clinical procedures that indicate illnesses that have not per se been noted.

### The problem of self-reported conditions

In the United States, there are no comprehensive, clinical record-based datasets for all ages and regions. Even the robust US Medicare databases do not currently have diagnostic data for people in managed care programs, and Medicare does not generally cover dental care (26). This problem is exacerbated by the frequent use of multiple, independent health care providers. In part because of these gaps in health data, federal and other health surveys employ self-reported conditions. These methods have both strengths and weaknesses (27), but they will never provide the fine detail and accuracy on personal conditions and medical procedures needed for full exploration of MCC.

What diseases and conditions should be included in the study of MCC? A remaining issue is that given the thousands of rubrics in the ICD, which are most important. The DHHS has selected 21 chronic conditions to begin emphasizing the problem (1). These are important conditions with public health implications. Many of the most serious and fatal conditions in Western societies are included in this list, but many other important conditions are not. While thoughtful prioritization is programmatically necessary, it may ignore the large number of less common diseases that cause great personal and family suffering. In the future, other priority lists may select diseases based on maximal preventability or the most application to diverse public health programs. Also, the increasing use of electronic health records, including narrative text, should enhance the ability to detect and evaluate large numbers of MCC (21).

Work on the application of MCC is proceeding rapidly. Alternative conceptualizations of MCC have been offered (28). Health care clinics and systems have been working to integrate the multimorbid patient into both primary and specialty care (29–31), including geriatrics and palliative care (32,33). More attention is being paid to the role of appropriate outcome measures sensitive to the MCC patient (19). The necessity of applying existing and newly developed clinical guidelines to the challenges of MCC is also being appreciated (34). Identifying the implications of MCC for health profession education is also occurring (33).

In the end, however, the value of collecting data on MCC, as with all scientific information, depends on how it will be used: whether it is understanding the natural history of diseases, applying clinical preventive interventions, identifying high-risk populations, making clinical or administrative decisions, counseling patients, or planning and evaluating large public health and prevention policies. It appears that much of what we know about health today derives from studying 1 disease at a time. Only recently has this begun to change. Public health has been a leader in addressing the clustering and effect of diverse conditions and MCC. For public health, the challenge will be to define preventive strategies that effectively deal with MCC, both before and after the doctor comes. Most challenging of all may be developing preventive strategies in the community that can favorably alter long-term clinical outcomes, and perhaps and hopefully alter the risk of acquiring multiple conditions after the first one has occurred.

## Some Methodological Suggestions for Addressing MCC Analyses

This collection of articles demonstrates the richness of federal databases for understanding the occurrence and clustering of MCC; this commentary highlights important challenges in organizing and interpreting MCC findings. This important work suggests some methodological steps when considering MCC analyses:

1. Clearly specify the research and programmatic questions and their relevance to public health and clinical practice.
2. Clearly specify all assumptions and definitions used in identifying chronic conditions and their associated risk factors and outcomes.
3. Identify and use the most relevant data to address the questions at hand; this may require using multiple data systems.
4. Attempt to incorporate the patient’s views of illness and suffering into the interpretation of MCC data.
